# INSOMNIA AND UNHEALTHY ALCOHOL USE DURING THE COVID-19 PANDEMIC AMONG WOMEN VETERANS AGE 50 YEARS AND OLDER

**DOI:** 10.1093/geroni/igad104.2689

**Published:** 2023-12-21

**Authors:** Caitlan Tighe, Deirdre Quinn, Karley Atchison, Rachel Bachrach

**Affiliations:** Providence College, Providence , Rhode Island, United States; VA Pittsburgh Healthcare System, Pittsburgh, Pennsylvania, United States; PIttsburgh VA Medical Center, Pittsburgh, Pennsylvania, United States; VA Pittsburgh Healthcare System, Pittsburgh, Pennsylvania, United States

## Abstract

Older women Veterans have a high burden of comorbid medical and mental health conditions and may be particularly at risk for co-occurring insomnia and unhealthy alcohol use. Some research suggests rates of both increased during the COVID-19 pandemic; however, few studies extended beyond pandemic year one or examined administrative data. Using Department of Veterans Affairs (VA) electronic health record data, we examined rates of insomnia (diagnoses, prescription insomnia medications) and unhealthy alcohol use (diagnoses, positive alcohol screens) in a national sample of women Veterans aged 50 years and older (N=239,638) prior to and during the pandemic (March 2018-2022). During this timeframe, insomnia rates increased across age groups. Annual insomnia rates were highest among women ages 50-59 (12.8-14.9%) and lower for women age 60 and older (6.2-12.1%). The annual rate of unhealthy alcohol use was also highest among women ages 50-59 (13.9-15.3%) and lower for women age 60 and older (5.4-12.9%). For the youngest and oldest age groups, rates of unhealthy alcohol use decreased annually until 2021 and then increased; for women aged 60-79, rates increased except in 2020, when rates declined. The proportion of women with both conditions in a given year ranged from .5-2.9% and was highest at younger ages and lowest at older ages. Together, results indicate annual rates of insomnia and unhealthy alcohol use during COVID were highest in midlife women but increased over time across ages, signaling a potential need for intervention and prevention efforts aimed at addressing sleep health and alcohol use across mid-late adulthood.

